# In vitro effects of lapachol and β-lapachone against Leishmania amazonensis

**DOI:** 10.1590/1414-431X2023e12693

**Published:** 2023-05-29

**Authors:** Á.C.F.H. Ramos-Milaré, B.G. Sydor, A.Á. Brustolin, D.S.S.L. Lera-Nonose, J. Oyama, E.L. Silva, W. Caetano, K.S.S. Campanholi, I.G. Demarchi, T.G.V. Silveira, M.V.C. Lonardoni

**Affiliations:** 1 Programa de Pós-graduação em Ciências da Saúde, Universidade Estadual de Maringá, Maringá, PR, Brasil; 2 Programa de Pós-graduação em Biociências e Fisiopatologia, Universidade Estadual de Maringá, Maringá, PR, Brasil; 3 Departamento de Química, Universidade Estadual de Maringá, Maringá, PR, Brasil; 4 Departamento de Análises Clínicas, Universidade Estadual de Maringá, Florianópolis, SC, Brasil; 5 Departamento de Análises Clínicas e Biomedicina, Universidade Estadual de Maringá, Maringá, PR, Brasil

**Keywords:** Leishmaniasis, Cutaneous leishmaniasis, Leishmania, Naphthoquinones, Natural products

## Abstract

Leishmaniasis is a neglected disease that affects millions of people worldwide, and special attention should be given to treatment because the available drugs have limitations, which can lead to low therapeutic adherence and parasitic resistance. This study evaluated the activity of the bioactive naphthoquinones, lapachol and β-lapachone, against *Leishmania amazonensis*. The cell alterations were evaluated *in vitro* on promastigote and amastigote forms. The lethal dose (LD_50_) at 24, 48, and 72 h on the promastigote's forms using lapachol was 75.60, 72.82, and 58.85 μg/mL and for β-lapachone was 0.65, 1.24, and 0.71 μg/mL, respectively. The naphthoquinones significantly inhibited the survival rate of *L. amazonensis* amastigotes at 83.11, 57.59, and 34.95% for lapachol (82.28, 41.14, and 20.57 µg/mL), and 78.49, 83.25, and 80.22% for β-lapachone (3.26, 1.63, and 0.815 µg/mL). The compounds on the promastigote's forms led to the loss of mitochondrial membrane potential, induced changes in the integrity of the membrane, caused damage to cells suggestive of the apoptotic process, and showed inhibition of tumor necrosis factor (TNF)-α and interleukin (IL)-6 production. The results showed that these naphthoquinones are promising candidates for research on new drugs with anti-*Leishmania* activity derived from natural products.

## Introduction

Leishmaniasis is a neglected tropical parasitic disease, which occurs mostly in developing countries and receives little investment. This disease is endemic in 98 countries and territories, with more than 350 million people at risk (1). The transmission occurs by inoculation of metacyclic promastigote forms through bites from infected female phlebotomine sandflies of the genus *Lutzomyia*. Cells of the mononuclear phagocytic system are where the parasite survives and replicates, so parasite elimination by macrophages is critical for host resistance to disease progression (2).

Cutaneous leishmaniasis (CL) is a polymorphic skin and mucosal disease causing single or multiple ulcerative or nodular wounds. It is considered a significant public health problem in some countries due to the several species of parasite and vectors and geographic differences involved. One of the main species responsible for CL is *Leishmania amazonensis*, the etiological agent of the diffuse, most severe and destructive clinical form of the disease, usually involving resistance and therapeutic failure (3,4).

Treatment is based on the use of a pentavalent antimonial drug and amphotericin B, as a second treatment choice. The available drugs are administered parenterally and are associated with several adverse effects and therapeutic failure, resulting in patients' abandonment of treatment (5). Research into new natural compounds with potential anti-*Leishmania* activity has increased and is essential for the development of new therapeutic strategies with lower toxicity and better accessibility (6-8).

Tabebuia avellanedae Lorentz ex Griseb, a popular tree known as “taheebo”, “lapacho”, “pau d'arco”, and “ipê roxo”, is the botanical nomenclature to specimens belonging to the genus *Tabebuia*, the largest genus of the Bignoniaceae family. It has been reported that *Tabebuia avellanedae* Lorentz ex Griseb contains furanonaphthoquinones, quinones, naphthoquinones, benzoic acid, benzaldehyde derivatives, cyclopentene dialdehyde, flavonoids, iridoids, and phenolic glycosides, compounds that may have pharmacological properties (9,10).

Plant-derived quinones and their derivatives may exert multifactorial effects on certain diseases. Due to their structural properties, they participate in biological oxidative processes, the redox property being one of their fundamental characteristics (11,12). Lapachol was known from the earliest reports as the most abundant quinone compound in the Bignoniaceae family (10). Lapachol is an important compound of the naphthoquinones class, which has been related to antiviral, antiparasitic, antimicrobial, anti-inflammatory, analgesic, anticancer, anti-metastatic, fungicide, and antioxidant activities (13,14).

β-lapachone, also known as *o*-naphthoquinone, is a natural constituent of specimens belonging to the *Tabebuia* genus or can be synthesized by acidic treatment of lapachol (15). It has biological activities including antibacterial, antiparasitic, anti-inflammatory, and anticancer activities (14,16,17).

The potential effects of lapachol and β-lapachone on *L. amazonensis* could be further investigated, and there are studies reporting that quinones and derivatives have anti-*Leishmania* activity (16,18-24). Considering that *in vitro* research of the possible activities of lapachol and β-lapachone on *L. amazonensis* is necessary and important to understand the biological activities of naphthoquinones and their possible therapeutic potential, the purpose of this study was to determine the leishmanicidal activity of lapachol and β-lapachone on *L. amazonensis in vitro*.

## Material and Methods

### Drugs and reagents

Drugs and reagents used in this study included the following: lapachol (4-hydroxy-3-(3-methylbut-2-enyl)naphthalene-1,2-dione); β-lapachone (2,2-dimethyl-3,4-dihydrobenzo[h]chromene-5,6-dione); amphotericin B (AmB) (Cristália^®^, Brazil); penicillin G (Sigma-Aldrich^®^, USA); streptomycin (Sigma-Aldrich^®^); 2,3-bis (2-methoxy-4-nitro-5-sulphophenyl)-2H-tetrazolium-5-carboxanilide (XTT) (Sigma-Aldrich^®^); phenazine methosulfate (PMS) (Sigma Chemical Co., USA); dimethyl sulfoxide (DMSO) (Sigma-Aldrich^®^); triton X-100 (Sigma-Aldrich^®^); Rhodamine 123 (Rh123, Sigma-Aldrich^®^); annexin V/propidium iodide (PI) (Sigma-Aldrich^®^); BD CBA Mouse Th1/Th2/Th17 Cytokine kit (BD Biosciences, USA); RPMI 1640 medium (Gibco^®^, USA); Medium 199 (Gibco^®^); fetal bovine serum (FBS) (Gibco^®^); Fast panoptic (Laborclin^®^, Brazil); Entellan (Merck^®^, Germany); trypan blue (Sigma-Aldrich^®^).

### Preparation of the compounds

**Figure 1:**
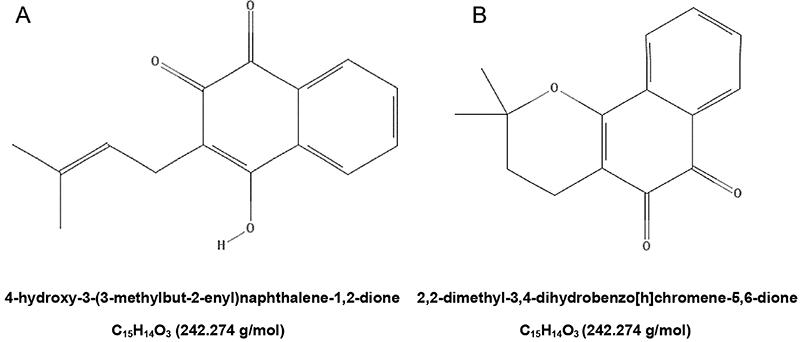
Structural formula of the compounds lapachol (**A**) and β-lapachone (**B**) derived from *Tabebuia avellanedae*.

Lapachol (Figure 1A) and β-lapachone (Figure 1B) used in the entire study were characterized in the Chemistry Laboratory of the State University of Maringá (UEM), Paraná, Brazil (25). Lapachol was extracted from the bark of *Tabebuia avellanedae* Lorentz ex Griseb and β-lapachone was obtained through an acid treatment (15). A specimen has been deposited at the Herbarium of the State University of Maringá, Brazil (*Tabebuia avellanedae* Lor. ex Griseb.: Brasil: Paraná: Maringá, Campus da Universidade Estadual de Maringá, (fr), J.H.G. Oliveira 10323 HUM, [lat: -23.4253005981445 long: -51.9385986328125 err: ±19250 WGS84]). The purity of the β-lapachone was calculated to be >98% by HPLC (25). Stock solutions of lapachol and β-lapachone were prepared aseptically in DMSO and diluted in culture medium (RPMI 1640 for amastigotes assay or 199 culture medium for promastigotes assay) at the time of use so that the DMSO concentration did not exceed 1% in the experiments. AmB was purchased commercially and used as the positive control of leishmanicidal activity.

### Ethical approval

All procedures performed in the studies involving animals were approved by the Ethics Committee on the Use of Animals of the UEM - CEUA under the number 3018070817/2017. The female BALB/c mice (8 weeks old) were obtained from the central animal house of UEM, Brazil.

### Macrophages

The *BALB/c* peritoneal macrophages were obtained by washing the peritoneal cavity with 8 mL of RPMI 1640 culture medium (26). The macrophage-like cell line J774A.1 (ATCC: TIB67, Banco de Células do Rio de Janeiro, Brazil) were cultured in RPMI 1640 medium with antibiotics (100 IU/mL penicillin and 0.1 mg/mL streptomycin), supplemented with 20% FBS in pH 7.6, and incubated at 37°C in 5% CO_2_. Trypan blue was used to analyze cell viability, and the experiment was carried out if viability was over 70%.

### Parasite strain and culture

*L. amazonensis* (MHOM/BR/1977/LTB0016) was maintained using animal infections, and the cultures were maintained by periodic subculture at 25°C (27). Briefly, BALB/c mice between 30 and 40 days of age were infected by inoculating 1×10^7^ parasites into the left footpad. After 30 to 40 days, the animals were anesthetized with isoflurane and then euthanized with CO_2_ in an appropriate chamber. Popliteal ganglions were removed and processed, and fragments were incubated in 199 culture medium supplemented with 10% FBS and antibiotics (100 IU/mL penicillin and 0.1 mg/mL streptomycin) at pH 7.2.

### Viability assay

The viability of *L. amazonensis* (MHOM/BR/1977/LTB0016) promastigotes was evaluated using the XTT colorimetric method, which consists in the XTT salt degradation by the mitochondria forming formazan crystals. Promastigote forms (2×10^7^ parasites/mL) from a culture in logarithmic growth phase were incubated with lapachol and β-lapachone diluted in 199 culture medium. The drugs were diluted in series, in a ratio of two, starting from 1/2, on a 96-well cell culture plate (TPP™ test plate, Switzerland). The compounds were tested at concentrations from 204.4 to 0.40 µg/mL (lapachol) and 157.5 to 0.31 µg/mL (β-lapachone). After 24, 48, and 72 h of incubation at 25°C, 100 μL of the XTT solution (500 μg/mL) activated with PMS (50 μg/mL) were added to each well and incubated for 4 h at 37°C. The result was measured by absorbance, determined by spectrophotometry (450/650 nm filter, VersaMax™ ELISA microplate reader, USA). The lethal dose (LD_50_) was defined as the concentrations of the compounds that reduced survival of *L. amazonensis* by 50% compared to untreated parasites in culture medium. AmB (1.96 to 0.25 µg/mL) was used as positive control of leishmanicidal activity. The therapeutic selectivity index (TSI CC_50_/LD_50_) was calculated by the ratio of toxicity to macrophage *vs* toxicity to parasites after 24 h incubation (28). All assays were performed in triplicate and repeated at least three times.

*Leishmania* growth was evaluated after 24 h of incubation at 25°C with the compounds under the same conditions as previously mentioned. Then, 10 µL of the content of each well was added to a 990 µL (1/100) of formalin solution (2%), and the parasites were counted in a Neubauer chamber. The inhibitory concentration (IC_50_) of promastigotes was calculated by comparing with untreated promastigotes in culture medium. The results were evaluated by linear regression of the inhibition percentage. The selectivity index (SI CC_50_/IC_50_) was calculated by the ratio of toxicity concentration to macrophage *vs* inhibitory concentration to the parasite growth after 24 h incubation (27). All assays were performed in triplicate and repeated at least three times.

### Activity on intracellular amastigotes

The activity of lapachol and β-lapachone on intracellular amastigotes was made on peritoneal macrophages (5×10^5^ per well). Cells were seeded onto round glass coverslips within 24-well plates in RPMI 1640 medium and incubated for 2 h at 37°C in 5% CO_2_. After that, the proportion of 6 parasites (*L. amazonensis)* per macrophage were added, and cultures were incubated for 4 h at 37°C in 5% CO_2_. Free parasites were removed by washing with RPMI 1640 medium. The infected macrophages were incubated with lapachol (82.28, 41.14, and 20.57 µg/mL) and β-lapachone (3.26, 1.63, and 0.815 µg/mL) for 24 h at 37°C. After the incubation time, the coverslips were removed, washed, stained with Fast Panoptic, and fixed on glass slides with Entellan. For the determination of cytokines, the supernatant was stored at −80°C. The experiments were done twice in triplicate, and treated uninfected cells were used as the cytotoxicity control and non-treated infected cells were used as the positive control of infection. The number of intracellular amastigotes was determined by counting 200 macrophages by concentration in an optical microscope (binocular biological microscope, mod. BA310E LED, Motic, Spain). The infection index was calculated by multiplying the percentage of infected macrophages by the number of parasites per macrophages (29).

### Macrophage cytotoxicity

Peritoneal macrophages from BALB/c mice were obtained as described before and adjusted to 1×10^6^ macrophages/mL and plated in 96-well culture plates (TPP™ test plate, Switzerland), followed by 2 h of incubation (5% CO_2_ at 37°C). J774A.1 macrophages were cultured in RPMI 1640 medium (Gibco), and during the experiment cell suspension adjusted for 5×10^5^ macrophages/mL was distributed in a 96-well culture plate (TPP™ test plate, Switzerland), followed by 48 h of incubation (5% CO_2_ at 37°C). In both cases, non-adherent cells were removed by washing with RPMI 1640 culture medium. After that, the compounds were tested with concentrations ranging from 408.75 to 0.03 μg/mL (Lapachol) and 630.0 to 0.04 μg/mL (β-lapachone). Cultures were incubated for 24 h at 37°C in 5% CO_2_, and non-treated cultures were used as viability control. The results were revealed using XTT colorimetric method (500 mg/mL activated with 50 mg/mL PMS, 100 μL/well of solution). After 4 h of incubation in 5% CO_2_ at 37°C protected from light, the results were measured as absorbance, determined by spectrophotometry (450/620 nm filter, VersaMax™ ELISA microplate reader) (28). The cytotoxicity concentration (CC_50_) was defined as the dose of the compound that reduced 50% of the survival of macrophages compared with untreated macrophages (30). At least three experiments were performed in quadruplicate.

### Change in cell volume of the parasites

Promastigote forms (2×10^7^ cells) of *L. amazonensis* untreated (negative control) or treated with 76.62 µg/mL of lapachol and 1.63 µg/mL of β-lapachone were analyzed to observe changes in cell morphology of the parasites induced by the treatment after 24 h of incubation. A validation of IC_50_ was made in an optical microscope using a Neubauer chamber, and observations were performed in EVOS cell imaging system using transmitted light (Thermo Fischer Scientific, USA); approximately 200 cells were observed in each condition using 1000 magnification. Subsequently, the parasites were analyzed using a BD FacsCalibur flow cytometer (Becton Dickinson, USA) and CellQuest Pro software (Joseph Trotter, Scripps Research Institute, USA), and 10,000 events were acquired. Dot plots were generated, the forward scatter (FSC-H) parameter was analyzed as presenting a correlation with the cell volume.

Scanning electron microscopy (SEM) also was performed to analyze morphological changes in cell surface topography. After 24 h of treatment, the parasites were centrifuged at 576 *g* for 10 min, and the samples were fixed in 2.5% glutaraldehyde and 0.1 M sodium cacodylate buffer. Poly-L-lysine was used to promote the adhesion of fixed parasites on coverslips. Promastigotes were dehydrated with increasing ethanol concentrations (30-100%), submitted to critical point drying, and metalized with gold for visualization on a Shimadzu 55-550 (Japan) microscope.

### Determination of the mitochondria membrane potential (Δ*Ψm*)

The Δ*Ψ*m was determined using on BD FacsCalibur™ flow cytometer and CellQuest Pro software, with a total of 10.000 events acquired, using a mitochondrial-specific green-fluorescent dye Rh123 (31). Briefly, *L. amazonensis* (2×10^7^ parasites/mL) were incubated with lapachol (76.62 µg/mL) and β-lapachone (1.63 µg/mL) for 1, 4, and 24 h at 25°C. After washed with PBS, the parasites were incubated with 5 μg/mL Rh123 for 15 min. After the incubation time, the cells were washed and suspended in PBS for additional incubation of 30 min for analysis. The percentage of fluorescence of Rh123, was determined by the (M_T_/M_C_)*100 equation, with MT representing the median fluorescence of the treated parasites and the MC the median fluorescence of negative controls. Alteration in Δ*Ψm* was quantified using an index of variation (IV) obtained by the equation IV = (M_T_ - M_C_)/M_C_, where negative IV values correspond to depolarization of the mitochondrial membrane. H_2_O_2_ (2 mM) was used as a positive control (32).

### Externalized phosphatidylserine in *L. amazonensis* promastigotes

*L. amazonensis* (2×10^7^ parasites/mL) were incubated with lapachol (76.62 µg/mL) and β-lapachone (1.63 µg/mL) for 24 h at 25°C. After treatment, the cells were centrifuged (901 *g* for 10 min) at room temperature and washed in PBS. According to the manufacturer's instructions, annexin V-FITC and PI (Sigma-Aldrich) were added. Data were acquired using the BD FacsCalibur™ flow cytometer (FL_1_ *vs* FL_2_) and CellQuest Pro software, with a total of 10,000 events acquired for analysis of results.

### DNA fragmentation assay

The presence of DNA fragmentation was evaluated by electrophoresis in agarose gel according to a previous study (33). *L. amazonensis* (1×10^6^ promastigotes/mL) was incubated with the IC_50_ of compounds for 24 h. After validation of IC_50_ as mentioned above, pellets were re-suspended in digestion buffer (10 mM ethylenediamine tetraacetic acid [EDTA], 50 mM Tris-HCl [pH 8.0] and 0.5% sodium lauryl sulfate), to which 0.5 mg/mL proteinase K was added. The mixture was incubated for 3 h in an ice bath and again for 1 h at 55°C in the presence of 0.1 mg/mL RNase A. DNA material was extracted by phenol/chloroform (1:1) treatment and was precipitated by adding 3 M sodium acetate and ice-cold ethanol (100%). After an overnight incubation at -20°C, the material was centrifuged (13270 *g*) for 30 min at 25°C temperature. Then, 2.5 volumes of ethanol (70%) were added to the pellet. The material was centrifuged (13270 *g*) for 15 min again and the pellet was collected, air dried, and re-suspended in Tris-EDTA buffer (50 µL, pH 8.0). DNA aliquots (10 µg) were electrophoresed on 2% agarose gel that contained ethidium bromide (0.5 µg/mL) using Tris-acetate-EDTA buffer (pH 8.0) for 45 min at 100 V and photographed under ultraviolet light. *L. amazonensis* untreated promastigotes were used as negative control of fragmentation (C-) and promastigotes treated with H_2_O_2_ as positive control (C+). The drag of the formed band was observed.

### Cytokine production

For the determination of cytokines, the supernatant of the amastigote assays stored at −80°C was used (MO, macrophages (negative control); MO + *L. amazonensis* (6 parasites/macrophage); samples treated with lapachol at IC_50_/2, IC_50_, and 2× IC_50_ with *L. amazonensis* (+) or without (-); the same was repeated for β-lapachone). The supernatant was centrifuged, and a 50 μL aliquot was used to determine cytokine levels (in *p*g/mL) using the BD Cytometric Bead Array (CBA) Mouse Th1/Th2/Th17 Cytokine kit (BD Life Sciences - Biosciences, USA). The dosage was performed in triplicate and according to the manufacturer's standards. This method is based on a suspension that contains beads, in which encoded fluorescent beads have cytokines that capture antibodies to bind the proteins. The final analysis was performed by flow cytometry using the BD FacsCalibur™ flow cytometer and CellQuest Pro software. The kit allows the measurement of seven distinct populations of Th1, Th2, and Th17 cytokines (IL-2, IL-4, IL-6, IFN-γ, TNF-α, IL-17A, and IL-10) in a single sample.

### Statistical analysis

The results are reported as means±SE or means±SD. For comparison between two groups with normal distribution, Student's *t-*test was used. When the data did not follow a normal distribution, the Mann-Whitney test was applied. In the analyses that included more than two groups, variance analysis was used, verifying the assumptions of independence, normality, and homoscedasticity of the residues. ANOVA followed by the Tukey *post hoc* test or Kruskal-Wallis followed by the Mann-Whitney pairwise test were conducted when the data did not follow the criteria described. The value of P<0.05 was considered statistically significant. Analyses were performed using BioEstat 5.0 software (free software, Manuel Ayres) and Past 3.X software (University of Oslo, Norway).

## Results

The naphthoquinones lapachol and β-lapachone (Figure 1) at low concentrations presented significant activity against promastigote and amastigote forms of *L. amazonensis*. After the treatment in promastigotes forms, alterations in the morphology, cell and nuclear integrity, mitochondrial membrane potential, and cytokine detection were shown.

### Viability assay

**Figure 2:**
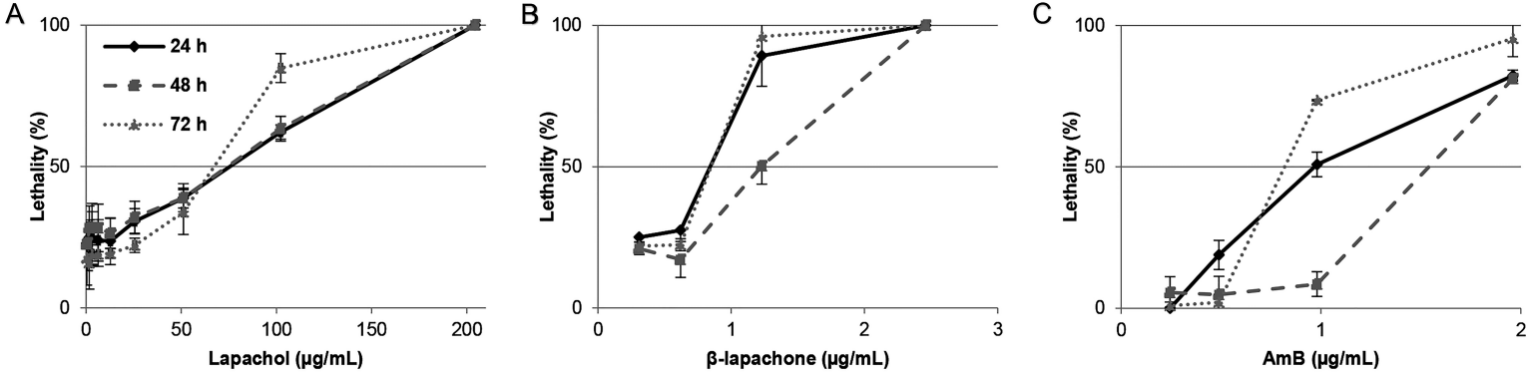
Activity of lapachol and β-lapachone against *Leishmania* promastigotes. *Leishmania amazonensis* promastigotes were incubated for 24, 48, and 72 h. **A**, lapachol was tested at concentrations from 204.4 to 0.40 µg/mL; **B**, β-lapachone was tested at concentrations from 157.5 to 0.31 µg/mL; **C**, AmB was tested at concentrations from 1.96 to 0.25 µg/mL (reference drug). The percentage of lethality was obtained using the XTT colorimetric method. Data are reported as means±SD of three replicates and are representative of three experiments.

Lapachol and β-lapachone induced *L. amazonensis* promastigote death (Figure 2). After 24, 48, and 72 h of incubation, the LD_50_ of lapachol was 75.60±6.12 µg/mL, 72.82±12.99 µg/mL, and 58.85±6.43 µg/mL, respectively (Figure 2A). The LD_50_ of β-lapachone was 0.65±0.18 µg/mL (24 h), 1.24±0.19 µg/mL (48 h), and 0.71±0.03 µg/mL (72 h) (Figure 2B). The LD_50_ of AmB was 0.97±0.09 µg/mL (24 h), 1.65±0.02 µg/mL (48 h), and 0.80±0.03 µg/mL (72 h) (Figure 2C). For all periods of incubation, β-lapachone showed an LD_50_ lower than that of lapachol (P<0.05 by ANOVA and Tukey *post hoc* tests). Comparing β-lapachone with AmB, there was a significant difference between LD_50_ at 24 and 48 h (P<0.05 by ANOVA and Tukey *post hoc* tests), which did not occur between lapachol and AmB. Lapachol and β-lapachone also showed an inhibitory effect on the growth of *L. amazonensis* promastigotes after 24 h of incubation, with concentrations that inhibited 50% of the promastigotes (IC_50_), ranging from 76.62±1.21 µg/mL for lapachol to 1.63±0.09 µg/mL for β-lapachone (Table 1).

**Table 1:** *In vitro* activity of lapachol and β-lapachone against promastigotes of *Leishmania amazonensis* and murine lineage macrophages.

Compounds	Official name	Promastigotes LD_50_(µg/mL)	Promastigotes IC_50_(µg/mL)	Murine macrophage cytotoxicity CC_50_(µg/mL)	J774A.1 macrophage cytotoxicity CC_50_(µg/mL)	Selectivity index - Murine macrophage/ J774A.1 macrophage	Therapeutic selectivity index - Murine macrophage/J774A.1 macrophage
Lapachol (1)	4-hydroxy-3-(3-methylbut-2-enyl)naphethalene-1,2-dione	75.60±6.12	76.62±1.21	41.14±2.17	26.66±8.69	0.54/0.35	0.54/0.35
β-lapachone (2)	2.2-dimethyl-3,4 dihydrodrobenzo[h] chromene-5,6-dione	0.65±0.18*^#^	1.63±0.09	3.21±2.72	3.18±0.04	1.97/1.95	4.94/4.89
Amphotericin B (AmB)	Amphotericin B	0.97±0.09	0.632±0.12	nd	nd	nd	nd

LD_50_: lethal dose for 50% of the promastigotes; IC_50_: inhibitory concentration for 50% of the promastigotes; CC_50_: cytotoxic concentration for 50% of macrophages; nd: not determined. Data are reported as means±SD. *P<0.05 between 2 and 1, ^#^P<0.01 between 2 and AmB (ANOVA followed by Tukey test).

### Activity on intracellular amastigotes

**Figure 3:**
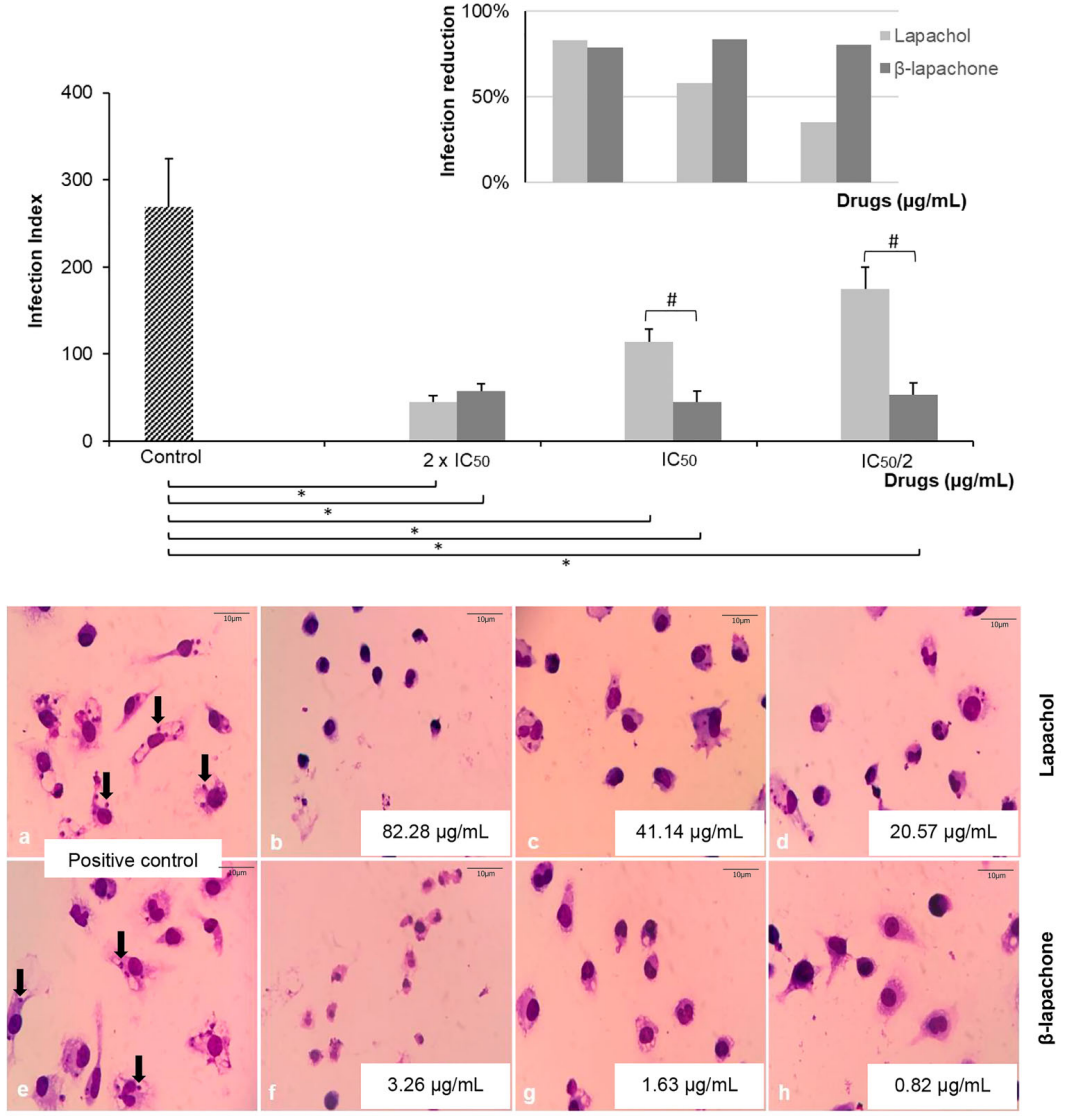
Effect of lapachol and β-lapachone derived from *Tabebuia avellanedae* on intracellular forms of *Leishmania amazonensis*. **Top figure**, the infection index was obtained by multiplying the percentage of infected macrophages by the average number of parasites per macrophage, and the percentage of infection reduction was obtained by subtracting from the control infection index the sample infection index and dividing this by the control infection index. The analysis was performed after 24 h of incubation (37°C and 5% CO_2_). ^#^P<0.05, comparing lapachol and β-lapachone using Student's *t*-test; *P<0.05 comparing the compounds to the control (untreated cells) using ANOVA followed by the Tukey test. **Bottom figure**, peritoneal macrophages from BALB/c mice were infected and treated with compounds. Infected macrophages without treatment (positive controls of infection: **a** and **e**). Arrows indicate amastigotes internalized in vacuoles. Infected macrophages treated with lapachol at 82.28, 41.14, and 20.57 µg/mL (**b**-**d**, respectively). Infected macrophages treated with β-lapachone at 3.26, 1.63, and 0.82 µg/mL (**f**-**h**, respectively). Changes in shape, size, and quantity of cells and amastigotes can be observed. All of the conditions were tested in triplicate and in two independent experiments using 1000× magnification (scale bar 10 μm). IC_50_: inhibitory concentration.

The naphthoquinones significantly inhibited the infection rate of *L. amazonensis* amastigotes in 83.11, 57.59, and 34.95% for lapachol and in 78.49, 83.25, and 80.22% for β-lapachone, respectively, at the concentrations tested (Figure 3). The results were obtained comparing infected macrophages treated with the positive control of infection.

### Cytotoxicity assay

The concentrations for cytotoxicity to 50% peritoneal macrophages (CC_50_/24 h) were 41.14±2.17 µg/mL for lapachol and 3.21±2.72 µg/mL for β-lapachone. The index for J774A.1 lineage macrophages ranged from 26.66±8.69 µg/mL for lapachol to 3.18±0.04 µg/mL for β-lapachone (Table 1). No statistical difference was found between the CC_50_ of lapachol and β-lapachone, as well as between both cell cultures tested (P>0.05 by Mann Whitney and Kruskal-Wallis test). Comparing TSI and SI of the compounds for murine macrophages, it was observed that β-lapachone was 3.65 times more selective for inhibiting the growth of *Leishmania* culture than lapachol, and 9.15 times more selective for *Leishmania* death. Similar results were observed for J774A.1 macrophages, in which β-lapachone was 5.6 times more selective for inhibiting the growth of *Leishmania* culture than lapachol and 14 times more selective for *Leishmania* death.

### Change in parasite cell volume

**Figure 4:**
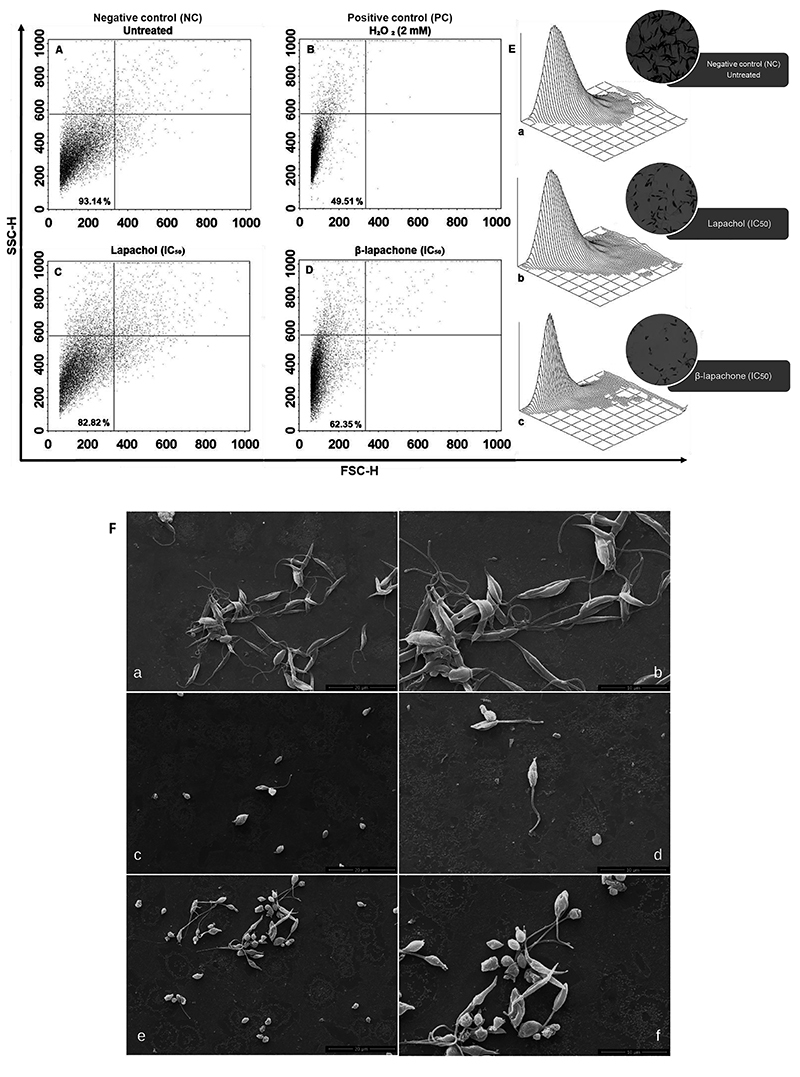
Cell volume of promastigote forms of *Leishmania amazonensis* treated with lapachol and β-lapachone. *Leishmania amazonensis* (2×10^7^ parasites/mL) untreated (**A**) and treated with lapachol and β-lapachone at concentrations of 76.62 and 1.63 µg/mL, respectively for 24 h (**C** and **D**). H_2_O_2_ (2 mM) was used as a positive control (**B**). The parasites were analyzed by means of a BD FacsCalibur flow cytometer and CellQuest Pro software, and 10,000 events were acquired in the region that corresponded to the parasites. **E**, Three-dimensional representative dot plots (**a**, **b**, and **c**) associated with images of promastigote forms (EVOS cell imaging system, 1000 magnification) used for the cytometer reading (circles on the right). **F**, Cell morphology by scanning electron microscopy: (**a** and **b**) negative control; (**c** and **d**) lapachol: 76.62 µg/mL; (**e** and **f**) β-lapachone: 1.63 µg/mL at 24 h (left column scale bar: 20 µm; right column scale bar: 10 µm). FSC-H: cell size. SSC-H: cell granularity. Typical dot-plots of at least two independent experiments.

Aiming to evaluate the influence of compounds on the morphology of the parasites, we used SEM and optical microscopy and EVOS cell imaging system using transmitted light. We found the occurrence of morphological changes in *L. amazonensis* promastigotes that were treated with lapachol and β-lapachone. Figure 4F (c-f) shows that cells treated with this compound exhibited loss of cell volume, rounder shape, flagellar alterations, and cellular shrinkage, compared to the untreated control, in which the promastigotes maintained the elongated form and exhibit only one flagellum (Figure 4F [a, b]). These findings observed in parasites were confirmed by flow cytometry analyzing the FSC-H parameter, which is related to cell volume, and the SSC-H parameter, which is related to cell granularity. Untreated promastigotes showed the percentage of gate cells at 93.14% (Figure 4A, lower left quadrant). After 24 h of treatment with 76.62 µg/mL of lapachol and 1.63 µg/mL of β-lapachone, marked decreases were observed in the percentage population of the lower left quadrant gate (82.82 and 62.35%, respectively; Figure 4C and D). The same was observed in the three-dimensional dot plot (Figure 4E [a-c]). Marked reductions (49.51%) were also shown in H_2_O_2_-treated cells used as a positive control of parasite cell volume change (Figure 4C).

### Determination of the mitochondria membrane potential (Δ*Ψm*)

**Figure 5:**
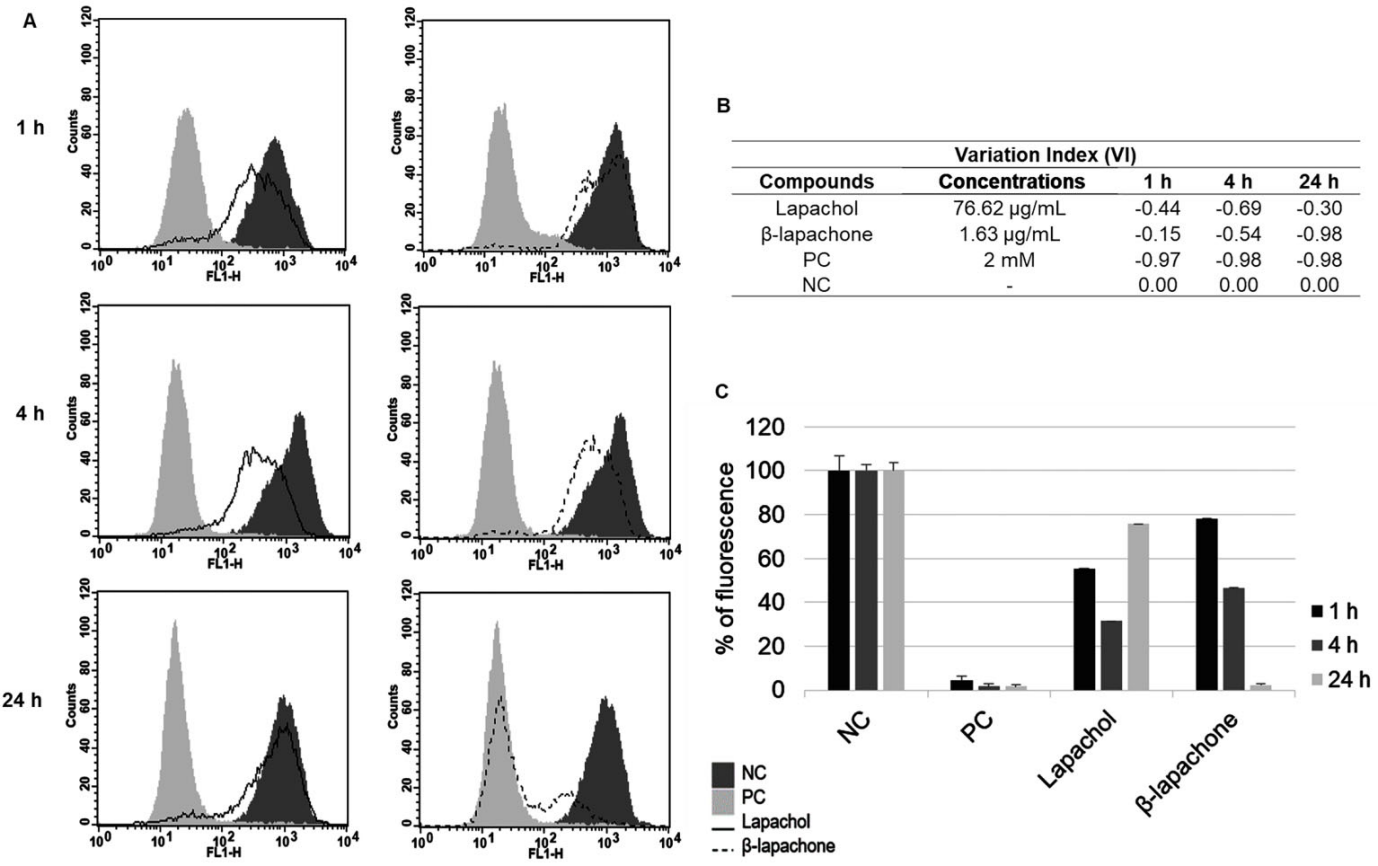
Alteration in the mitochondria membrane potential of *Leishmania amazonensis* after treatment using lapachol and β-lapachone. **A**, Representative histograms showing treated promastigotes stained with Rh123 by flow cytometry. **B**, Variation index (VI) values were obtained by the (MT−MC)/MC equation. **C**, Graphic representation of fluorescence intensity percentage of the negative control (NC: untreated cells), positive control (PC: H_2_O_2_, 2 mM), and different treatment groups. Typical histograms of at least three independent experiments. Data are reported as means and SD.

The fluorescent probe rhodamine 123 was used to evaluate the mitochondrial function of the parasites by flow cytometry assay. Both compounds caused a marked decrease in total Rh123 fluorescence intensity after treatment for 24 h, indicating mitochondrial depolarization compared to untreated parasites, represented by the histogram of total Rh123 fluorescence (Figure 5A), variation index (Figure 5B), and percentage of fluorescence intensity (Figure 5C). The reduction in Δ*Ψm* of lapachol (76.62 µg/mL) was observed already at 1 h (44%), which increased at 4 h (69%) and decreased at 24 h (30%). The loss of mitochondrial potential was more pronounced for β-lapachone (1.63 µg/mL), which occurred concurrently with a longer time of exposure to treatment (15%, 1 h; 54%, 4 h; 98%, 24 h). The value observed at 24 h for β-lapachone was similar to values found in the positive control (H_2_O_2_), which induced 97, 97, and 98% of changes in the Δ*Ψm* in parasites after 1, 4, and 24 h of treatment (Figure 5B).

### Externalized phosphatidylserine in *L. amazonensis* promastigotes

To determine whether the effect of lapachol and β-lapachone on *L. amazonensis* occurred because of the induction of apoptosis, cells treated with the compounds were investigated for annexin V-FITC/PI labelling. The promastigote forms treated with lapachol (76.62 µg/mL) and β-lapachone (1.63 µg/mL) for 4 h showed annexin labeling (AnV+, PI-) of 8.57 and 9.03%, respectively (Supplementary Figure S1). After 24 h of incubation (Supplementary Figure S1), it was not possible to observe the increase of this process, so it should be appropriately tested using longer incubations. The level of apoptosis after treatment was comparable to that triggered by the AmB, which presented the same behavior of the compounds.

### DNA fragmentation assay

**Figure 6:**
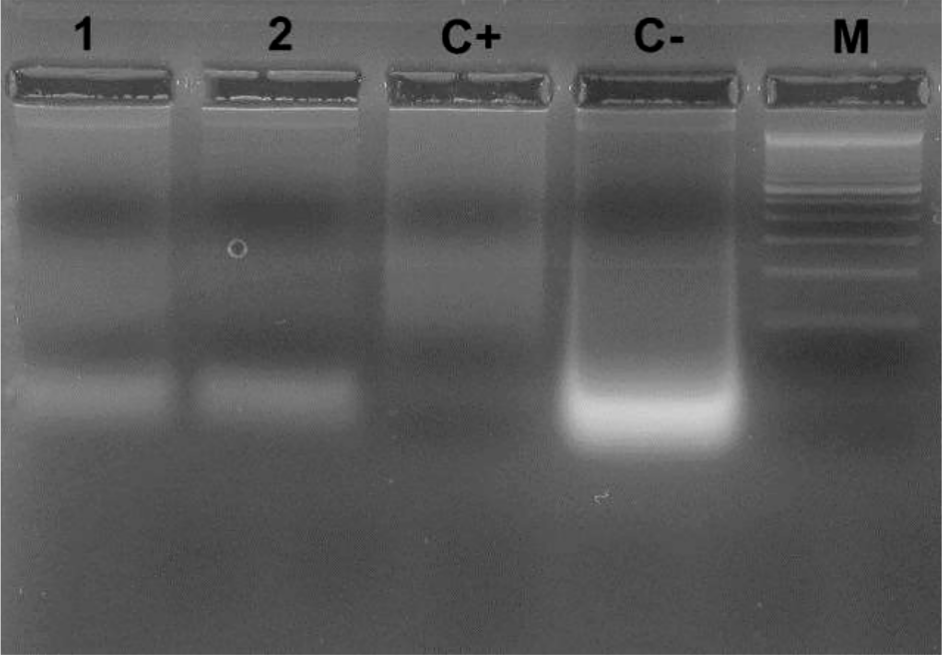
DNA fragmentation of *Leishmania amazonensis* induced by lapachol and β-lapachone. The fragmentation was observed in agarose gel electrophoresis. A DNA ladder marker (100 bp) is shown in lane M. Promastigotes of *Leishmania amazonensis* were untreated (C-), treated with H_2_O_2_ (C+), treated with 76.62 µg/mL (IC_50_) of lapachol (lane 1), and treated with 1.63 µg/mL (IC_50_) of β-lapachone (lane 2) for 24 h. The figure shows a representative result of two experiments.

*L. amazonensis* treated with the IC_50_/24 h of lapachol and β-lapachone for 24 h promoted DNA fragmentation (Figure 6). DNA fragments were observed in treated parasites and it was possible to notice different degrees of smearing between the treated and non-treated cultures (C-). The cultures treated showed fragmentation more similar to the positive control (C+).

### Cytokine production

**Figure 7:**
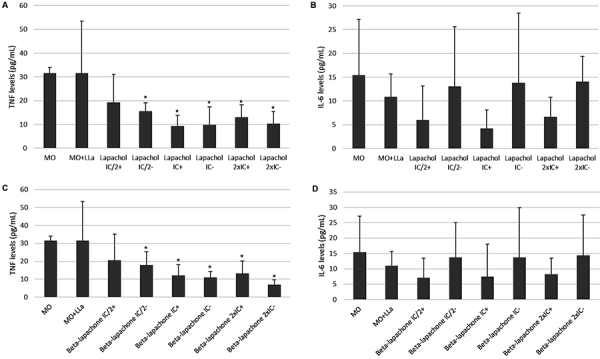
Immunomodulation of cytokine tumor necrosis factor (TNF) and interleukin-6 (IL-6) production in murine macrophages treated with lapachol (**A** and **B**), β-lapachone (**C** and **D**), and uninfected or infected with *Leishmania amazonensis.* The experiment was performed in triplicate. The incubation period was 24 h. Flow cytometry was performed to determine cytokine levels (pg/mL). MO: macrophages (negative control); MO+*LLa*: *L. amazonensis* (6 parasites/macrophage). Samples were treated with lapachol at inhibitory concentration (IC_50_)/2, IC_50_, and 2× IC_50_ with (+) or without (-) *LLa*. The same protocol was repeated for β-lapachone. *P<0.05, compared with uninfected macrophages (negative control) using ANOVA followed by the Tukey test.

Macrophages that were infected with *L. amazonensis* and treated with lapachol and β-lapachone did not present significant changes in IL-2, IL-4, IFN-γ, IL-17A, and IL-10 production. Differences were only found in the production of TNF-α (Figure 7A and C) and IL-6 (Figure 7B and D). Infected and untreated macrophages presented a decrease in TNF-α and IL-6. Uninfected and treated macrophages at all concentrations tested presented a decrease in TNF-α and IL-6 levels. The decrease was more evident when analyzed in infected and treated macrophages.

## Discussion

Lapachol and β-lapachone derived from *Tabebuia avellanedae* Lorentz ex Griseb promoted striking cell morphological alterations, mitochondrial depolarization, DNA fragmentation, decrease of the infection index in the amastigotes assay, and decrease of cytokines. These results suggested that the treatment could induce damage in parasites treated and modulate important cytokines of the immune system. Therefore, they are strong candidates for research on new drugs derived from natural products with anti-*Leishmania* activity, which has become an important source of drugs (6-8). Since the current available treatment presents limitations, including side effects, therapeutic failure, relapse, and recurrent cases (5), the development of drugs that are safe, effective, and that have lower toxicity have been increasingly required for the treatment of leishmaniasis.

This study showed the leishmanicidal effects of lapachol and β-lapachone against promastigote and amastigote forms of *L. amazonensis*. It is important to consider that the species of *Leishmania* investigated in the present study is related to the anergic diffuse cutaneous leishmaniasis (ADCL), which has a T cell hyposensitivity pole and a marked T helper type 2 immune response. These immune conditions can be related to therapeutic failure and parasite resistance (26).

Lapachol and β-lapachone at low concentrations presented significant activity against promastigote. The IC_50_ at 24 h of lapachol was 76.62 µg/mL (316.25 µM), and for β-lapachone was 1.63 µg/mL (6.73 µM). Guimarães et al. (23) also studied the activity of lapachol and β-lapachone but on different strains of *L. amazonensis* (strains MHOM/BR/1989/ BA199), and the IC_50_ found at 72 h was 102.05 µM for lapachol and 1.39 µM for β-lapachone. Moreno et al. (24) demonstrated the activity of β-lapachone against *Leishmania major* promastigotes, and the IC_50_ value after 48 h of incubation was 1.5±0.7 µM.

When the survival rate of *L. amazonensis* amastigotes was investigated, we observed that the compounds also had an action against the intracellular forms. This was an important result, as amastigotes forms are responsible for clinical manifestations in the vertebrate host and are the main target of chemotherapy for leishmaniasis. A decrease in the percentage of infected macrophages and in the number of intracellular parasites was observed. The infection reduction caused by β-lapachone was maintained similar in all tested concentrations, while lapachol demonstrated a dose-dependent action. Similar results with other naphthoquinone derivatives and different species or strain of *Leishmania* have been found (18,21,34). For example, Teixeira et al. (18) demonstrated that the effects of lapachol inhibited the infection rate of amastigotes of *L. (V.) braziliensis* (MHOM/BR/94/H-3227) by 88.8% using 50 µg/mL.

Usually, the process of cell damage is initiated by the transduction of stress signals originating from extracellular (extrinsic) or intracellular (intrinsic) sources, such as intracellular stress caused by drug treatment (35). To better understand the effects of lapachol and β-lapachone, we also evaluated cell alterations. The drugs reduced the cell size of *L. amazonensis* promastigotes after 24 h. Flow cytometry analysis and SEM showed decreased cell volume in lapachol and β-lapachone-treated promastigotes. Similar results were described by Mendonça et al. (34) when researching the activity of Flau-A, a naphthoquinone that induced morphology modifications on promastigotes of *L. amazonensis,* decreased their mobility, and altered the flagellum size, factors that were associated with possible alterations in mitochondrial activity.

Undoubtedly, the mitochondria are essential organelles in parasites. *Leishmania* protozoa have a single organelle in their cytoplasm and a mitochondrial dysfunction causes irreversible damage leading to cell death because there is no possibility of compensating for injured mitochondria (32,34). Our study showed that lapachol and β-lapachone act in this organelle. Flow cytometric assay using a Rh123 probe was used to determine the Δ*Ψm* on promastigotes of *L. amazonensis*. Considering that Rh123 is sensitive to Δ*Ψm*, membrane depolarization induces a loss of Rh123 fluorescence, whereas hyperpolarization induces an increase in fluorescence (36). Results showed that treatment affected the electrochemical gradient of the parasites' mitochondrial membrane during oxidative phosphorylation by reducing their Δ*Ψm*. Furthermore, we showed that β-lapachone had significantly better depolarization compared to lapachol, which demonstrated some repolarization after 24 h. In some cases, a transient hyperpolarization occurs before mitochondrial depolarization, although both hyperpolarization and loss of Δ*Ψm* may result in the death of promastigote forms, demonstrating the importance of maintaining Δ*Ψm* suitable for parasite survival (36).

Naphthoquinone derivatives have diverse biological properties and participate in multiple biological oxidative processes. Therefore, synthesis and design of novel compounds are increasing, with focus on improving the activity against the genus *Leishmania,* with a generation of novel molecules that inhibit cellular organelles/processes and increase reactive oxygen species (ROS) and lipophilicity to enhance penetration through the membrane (19).

The fundamental feature of quinones is their redox properties, which are induced through the formation of an aromatic system. Thus, the formation of reactive oxygen species (ROS) due to the direct reaction with proteins, lipids, and DNA causes oxidative stress, mitochondrial membrane depolarization, and DNA fragmentation (11,19). These are some of the processes that were reproduced in our experiments in this work.

As mentioned, mitochondrial dysfunction can culminate in cell death by apoptosis, and our results subtly suggested that the compounds exert leishmanicidal activity probably via apoptosis, since we observed cell damage, phosphatidylserine externalization, and DNA fragmentation in the results of the experiments. Exposure to phosphatidylserine is the hallmark of classic apoptosis, as this molecule is a phospholipid present on the inner surface of the cell membrane and its exteriorization occurs during the process of apoptosis. Annexin V is a molecule with affinity for phosphatidylserine, binding to it at the beginning of the apoptotic process. In contrast, PI binds to the DNA of cells in the final phase of apoptosis, when membrane integrity is already lost. Araújo et al. (37) also described results from the action of lapachol suggestive of an apoptotic process. The group analyzed the induction of phosphatidylserine exposure and cell-cycle arrest at sub-G0/G1 phase, confirming the apoptotic-like cell death in *L. amazonensis* promastigotes upon lapachol treatment.

The investigation of immune mechanisms in eliminating *Leishmania* parasites is important when we study a new molecule for treatment. In CL, host immunity is one of the most critical factors in disease diagnosis and cure (1). Infection control occurs through the activation of immune response cells, such as Th_1_ cells and macrophages that produce IFN-γ, TNF-α, IL-12, and other cytokines that promote the activation of microbicidal mechanisms and the killing of the parasite by macrophages. This study observed that macrophages infected with *L. amazonensis* and treated with lapachol and β-lapachone showed no significant changes in IL-2, IL-4, IFN-γ, IL-17A, and IL-10. However, differences were found in TNF-α and IL-6, and both cytokines were downregulated.

Interestingly, in Brazil, *L amazonensis* are the major agents of ADCL, a rare clinical form. Although ADCL is the most severe and incurable form of CL, it occurs in about 1% of CL cases each year. In ADCL, nodular skin lesions are rich in amastigote forms, and the parasite is disseminated throughout the body. This suggests a specific failure of protective immunological mechanisms to control infection. As a result, there is preferential activation of the Th_2_-type immune response, with the production of cytokines that negatively regulate the immune response and culminate with an inadequate response to traditional forms of treatment (4). Our results agreed with this because they showed inhibition of TNF-α and IL-6 production and the absence of stimulation of cytokines that are important in CL control, suggesting that other mechanisms are necessary to control the infection.

The present results strengthen the possibility of using lapachol and β-lapachone, a natural and a synthesized compound, respectively, in the treatment of CL. According to de Castro et al. (19) and our results, β-lapachone is the most promising bioactive molecule of the lapachol group. In general, the naphthoquinones tested had a considerable activity against amastigote forms of *L. amazonensis*, inducing several morphological changes on promastigotes, including alterations in mitochondrial potential, loss of membrane integrity, and probably cell damage by the apoptotic process. These data are an essential contribution to the preclinical studies of lapachol and β-lapachone, demonstrating the promising therapeutic action of these new molecules on leishmaniasis. Further investigation is necessary to characterize which additional mechanisms of action are involved with the effects of lapachol and β-lapachone.

## Supplementary Material

Click here to view [pdf].
